# Differences among Major Taxa in the Extent of Ecological Knowledge across Four Major Ecosystems

**DOI:** 10.1371/journal.pone.0026556

**Published:** 2011-11-02

**Authors:** Rebecca Fisher, Nancy Knowlton, Russell E. Brainard, M. Julian Caley

**Affiliations:** 1 Australian Institute of Marine Science, UWA Oceans Institute, Crawley, Australia; 2 Department of Invertebrate Zoology, National Museum of Natural History, Smithsonian Institution, Washington D.C., United States of America; 3 Center for Marine Biodiversity and Conservation, Scripps Institution of Oceanography, University of California San Diego, La Jolla, California, United States of America; 4 Coral Reef Ecosystem Division, Pacific Islands Fisheries Science Center, National Marine Fisheries Service, National Oceanic and Atmospheric Administration, Honolulu, Hawaii, United States of America; 5 Australian Institute of Marine Science, Townsville, Australia; Biodiversity Insitute of Ontario - University of Guelph, Canada

## Abstract

Existing knowledge shapes our understanding of ecosystems and is critical for ecosystem-based management of the world's natural resources. Typically this knowledge is biased among taxa, with some taxa far better studied than others, but the extent of this bias is poorly known. In conjunction with the publically available World Registry of Marine Species database (WoRMS) and one of the world's premier electronic scientific literature databases (*Web of Science*®), a text mining approach is used to examine the distribution of existing ecological knowledge among taxa in coral reef, mangrove, seagrass and kelp bed ecosystems. We found that for each of these ecosystems, most research has been limited to a few groups of organisms. While this bias clearly reflects the perceived importance of some taxa as commercially or ecologically valuable, the relative lack of research of other taxonomic groups highlights the problem that some key taxa and associated ecosystem processes they affect may be poorly understood or completely ignored. The approach outlined here could be applied to any type of ecosystem for analyzing previous research effort and identifying knowledge gaps in order to improve ecosystem-based conservation and management.

## Introduction

Existing knowledge shapes our understanding of ecosystems and determines our ability to identify what drives ecosystem function and promotes ecosystem resilience and understand the nature and role of keystone species. Such information is critical to the successful conservation of the world's biodiversity and increasingly underpins management, particularly the broad approach referred to as ecosystem-based management (EBM). However, while a large and growing body of ecological knowledge is stored in the scientific literature, representing a broad range of the world's ecosystems, this existing knowledge may not adequately represent the range of taxa present in these ecosystems. An understanding of this potential bias is becoming increasingly urgent as biodiversity is lost [1], ecosystems are degraded [2] and the vitally important goods and services that they provide are threatened [3], [4].

By concentrating on four major marine ecosystems, we examine the taxonomic distribution of existing ecological knowledge and the extent to which various taxonomic groups may be under- or over-represented in our knowledge of these systems. We analyzed the literature for coral reefs, seagrass beds, mangroves and kelp beds because these ecosystems provide important ecosystem goods and services both individually and via functional linkages [4], [5], [6], [7], [8], [9], [10]. In addition, each ecosystem is relatively discrete and therefore easy to delineate and is defined by its dominant habitat-forming organisms. Also, these ecosystems are at considerable risk from both direct and indirect anthropogenic pressures such as pollution, development, overfishing and now global warming and ocean acidification [11], [12]. Indeed, few if any areas remain where these ecosystems have not been impacted to some extent [11]. Therefore, now more than ever, it is important to assess our current knowledge of these ecosystems and consider how future research efforts may be best allocated to maximize our chance of achieving sustainable management. Using text mining (following [13]) of papers contained within the *Web of Science*® (WoS), we examined how existing ecological knowledge and associated research efforts are distributed among different taxonomic groups.

## Methods

### Sampling the scientific literature

*Web of Science*® (WoS) is one of the world's largest literature databases and includes much of the published information relevant to marine ecology. WoS was searched between the years 1957–2009 by using the following keywords: “coral reef/s”, “mangrove forest/s”, “kelp forest/s” “seagrass bed/s” and “seagrass meadow/s”. “Coral”, “mangrove”, “kelp” and “seagrass” were not used on their own as search terms. This was done to ensure that returns were relevant to the ecosystems of interest, rather than simply including all possible studies of these particular organisms. The resulting 13,229 papers were exported in EndNote® format and transferred to Microsoft Access®. Structure Query Language (SQL) was then used to further limit the resulting data to those containing these same search terms in the title, keywords (author keywords only [13]) or abstract fields. The filter resulted in a set of 9303 papers, less than the number produced by the WoS search, because it removed papers that refer only to these search terms in the KeyWords Plus® field [14], [15] or in other WoS fields not used here.

### Taxonomic assignment

Text from the title, keywords and abstracts was matched against scientific names contained within the World Registry of Marine Species (WoRMS [16]). To achieve this, the open source statistical programming language R [17] was used to generate a vector of all unique single and double word sequences from the text of the title, abstract and keywords, which was then matched against WoRMS. Research papers were limited to those that could also be assigned to a taxonomic group at the phylum level or better. For simplicity of interpretation, results were limited to the Animalia, Plantae and Chromista kingdoms; within the WoRMS database these kingdoms encompass all animals, plants (including red and green algae) and brown algae, respectively. Because taxonomic assignment was based entirely on the WoRMS database, the patterns observed here depend on the named taxa occurring therein. Valid species named in WoRMS are 87% checked by taxonomic editors and represent 87% of the estimated named marine species. WoRMS contains synonyms as well as valid taxonomic names. Papers containing a match to a taxonomic name listed as a synonym in WoRMS were assigned to the valid taxonomic name for that synonym.

Taxonomic names were searched only in the title, keywords and abstracts. Therefore, all relevant literature may not have been captured, particularly for ecological research where functional roles were the focus of titles and abstracts with specific taxa mentioned only in the text. Only some literature is available as full text in a searchable electronic format, thus making it difficult to expand the search beyond the fields we searched. In addition, full text will in many cases refer to taxa names that are not the focal species of a study, but are instead discussed to provide context for the results being reported. Rather than retrieving every publication that referred to a particular species, our goal was to develop a relative index of research effort. The taxonomic patterns in research effort reported here thus assume that the ratio of literature containing specific taxonomic names (in the titles, keywords and abstracts) relative to those that do not include such specific information in these fields are equivalent across major taxonomic groups. Furthermore, it is likely that ecological studies that do not include specific taxonomic information focus on better-known taxa; thus, any patterns of bias in research effort would be reinforced if this literature were also included.

### Analyses

The number of papers, classes and species occurring in the literature was calculated for each of these four ecosystems and within each phylum. Shannon's evenness [18] index at both the species and class levels was calculated as −∑*Pi* ln(*Pi*)/ln*S*, where *S* is the number of species and *Pi* is the proportion of total abundance of the *i*th species. Chao's [19] estimates of species richness and taxonomic distinctness [20] were also calculated using the vegan [21] package in R. Taxonomic distinctness is a measure of the average distance between all pairs of species in a taxonomic tree, which captures phenotypic differences and functional richness [20]. Taxonomic distinctness was calculated across the whole data set, as well as for three separate periods (prior to 2000, 2000–2006, 2006–2009). This selection of periods divided the literature into roughly equal-sized sample bins, allowing us to examine how the taxonomic breadth of research effort has changed through time. Individual-based rarefaction [22] was used to graphically examine species richness with increasing sample effort (number of papers) among the four ecosystems.

The numbers of papers within each phylum, class and species were calculated and frequency histograms were used to examine patterns in the number of papers within different classes across the four ecosystems. The probability of occurrence within the literature was estimated for each class by fitting binomial models using the function glm of the package stats [23] in R and equations detailed in [24]. To explore the relationship between research effort and global known richness of named species, we plotted the total number of research papers as a function of the total number of valid species contained within WoRMS. Trends in this relationship were analyzed using Generalized Additive Mixed Models (GAMM) [24] and were fitted using the function gamm in the mgcv [25] package in R. Both the number of papers and number of species were log_10_ transformed to remove “trumpeting” of variances. To remove some taxonomic non-independence, phylum was included as a random effect. Deviations (residuals) from these GAMMs were used as an estimate of species richness corrected by research effort for each class. To determine the most well-studied taxa, different classes were ranked based on the probability of occurrence in the literature across all four ecosystems, as well as research effort corrected for species richness as described above.

## Results

**Figure 1:**
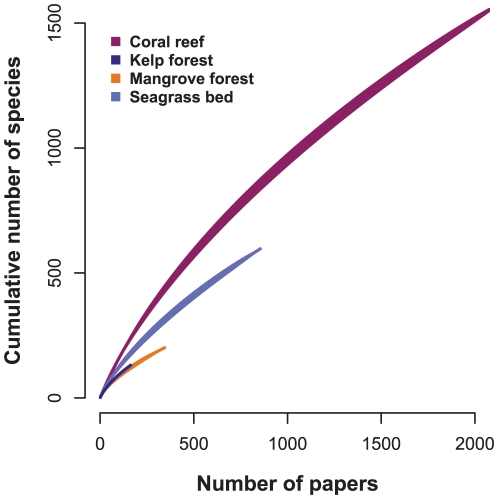
Species richness of taxa occurring in *Web of Science*® literature for four marine ecosystems. Shown are 95% confidence clouds of individual-based rarefaction curves [22] generated using the specaccum function in the vegan [21] package of R.

**Figure 2:**
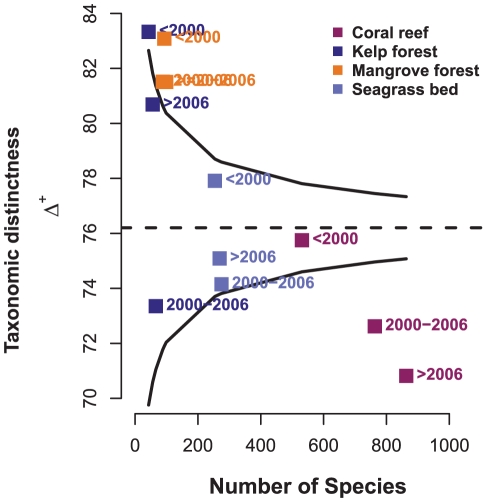
Taxonomic distinctness of *Web of Science*® literature for four marine ecosystems. Taxonomic distinctness (Δ^+^) plotted against the number of species for three time periods (prior to 2000, 2000–2006 and 2007–2009) across four marine ecosystems. The dashed line denotes the simulated mean and solid lines (funnel) indicate the approximate 2*standard deviation limits. Points falling outside the 2*standard deviation limit can be considered ‘significantly’ higher (greater taxonomic breadth present above) or lower (less taxonomic breadth present, below) than the simulated mean.

A total of 2380 unique species from 78 taxonomic classes of marine organisms of the kingdoms Animalia, Chromista and Plantae as defined by WoRMS were detected in the ISI indexed literature for these four major marine ecosystems (Table 1). This total represents 57% of the valid classes from these Kingdoms contained in WoRMS (Table 2). Coral reefs dominated in terms of the diversity of taxa studied, with at least one paper found for 1580 species from 66 classes (Table 1). Coral reefs were followed in diversity by 597 species in the seagrass bed literature (50 classes), 201 species in the mangrove forest literature (38 classes) and 131 species (22 classes) in the kelp forest literature. Chao's estimators of species diversity followed a similar pattern to raw species richness, with greatest diversity found in the coral reef literature, followed by literature on seagrass beds, mangrove forests and kelp forests (Table 1, Fig. 1). Patterns in taxonomic distinctness (a measure of the average distance between all pairs of species in the taxonomic tree) differed from species richness, with greatest distinctness occurring in the mangrove and kelp forest literatures, followed by the seagrass bed literature and finally with the coral reef literature being the least taxonomically distinct, indicating that a smaller range of taxonomic groups are well represented (Table 1, Fig. 2). Within each ecosystem, taxonomic distinctness was greatest for literature dating prior to 2000 and was less distinct or similarly distinct for the two more recent time periods (2000–2006 and 2006–2009). Across all ecosystems and time periods, the smallest value for taxonomic distinctness was found for the most recent research on coral reefs.

**Table 1:** Number of research papers, classes and species, and diversity occurring in *Web of Science*® indexed literature for four marine ecosystems.

		CR	KF	MF	SB
Counts	Papers	6535	322	1152	1557
	Classes	66	22	38	50
	Species	1580	131	201	597
Evenness	Class	0.56	0.66	0.54	0.61
	Species	0.91	0.80	0.84	0.80
Chao		3741±201	339±67	555±96	1703±171
Delta+		73.63	80.57	83.75	79.15

CR – Coral reefs, KF – Kelp forests, MF – Mangrove forests, SB – Seagrass beds. Values shown are the number of research papers (Papers), classes and species, and three diversity measures (Shannon evenness index, Chao estimates of species richness and taxonomic distinctness) based on taxa occurring in *Web of Science*® indexed literature for four marine ecosystems.

**Table 2:** Numbers of research papers by taxonomic class for four marine ecosystems.

Class - Common names	CR	KF	MF	SB	Total
Actinopterygii- Ray-finned fishes	**1256**	29	31	**243**	1559
Anthozoa - Anemones, corals (various)	**994**	7	2	11	1014
Liliopsida - Seagrasses	45	2	26	**553**	626
Malacostraca - Crabs, lobsters, shrimp, krill, amphipods, isopods	233	29	**90**	236	588
Magnoliopsida - Mangroves	34	0	**350**	17	401
Phaeophyceae - Brown algae (including kelp)	145	**122**	3	28	298
Gastropoda - Snails, slugs	102	26	50	47	225
Echinoidea - Sea urchins, sand dollars	115	**34**	2	39	190
Demospongiae - Sponges	159	0	6	24	189
Bivalvia - Bivalves	86	2	18	79	185
Florideophyceae - Red algae	124	9	4	33	170
Polychaeta - Segmented worms	68	5	11	49	133
Hydrozoa - Hydrozoans	109	3	4	4	120
Bryopsidophyceae - Green algae (various)	57	2	0	41	100
Asteroidea - Starfish	78	2	1	11	92
Ulvophyceae - Green algae (sea lettuce)	46	2	3	31	82
Gymnolaemata - Moss animals	77	1	0	2	80
Maxillopoda - Barnacles, copepods	40	2	12	23	77
Mammalia - Mammals	15	12	7	27	61
Ascidiacea - Sea squirts	30	2	0	5	37
Insecta - Insects	0	0	32	1	33
Holothuroidea - Sea cucumbers	20	0	0	13	33
Aves - Birds	4	2	15	10	31
Reptilia - Reptiles (sea snakes, turtles, crocodiles)	22	0	2	7	31
Tentaculata - Comb jellies (with tentacles)	29	0	0	0	29
Elasmobranchii - Sharks, rays, skates	22	0	1	5	28
Trematoda - Flukes	27	0	1	0	28
Monogenea - Ectoparasitic flatworms	20	0	1	1	22
Adenophorea - Roundworms	8	0	7	3	18
Scyphozoa - True jellyfish	16	0	0	0	16
Ophiuroidea - Brittle stars and basket stars	15	0	0	1	16
Thaliacea - Salps and relatives (all free-floating)	1	0	0	12	13
Bacillariophyceae - Pennate diatoms	4	0	3	4	11
Ostracoda - Seed shrimp	5	0	2	4	11
Chlorophyceae - Green algae (various)	6	1	1	2	10
Crinoidea - Sea lilies, feather stars	6	0	4	0	10

CR – Coral reefs, KF – Kelp forests, MF – Mangrove forests, SB – Seagrass beds. Class information was obtained from the World Registry of Marine Species (WoRMS [16]). Only classes with at least 10 occurrences in the literature indexed in *Web of Science*® for any of the four ecosystems are shown. A full list of all classes with at least 1 occurrence can be found in Table S1.The two most studied classes for each ecosystem are shown in **bold**. Common names are not comprehensive but provide examples for the groups; in some cases no common names specific for the group exist and more general common names are provided.

**Figure 3:**
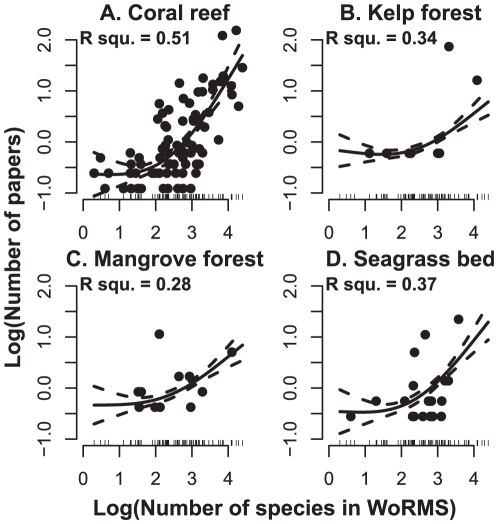
The relationship between the number of *Web of Science*® papers and currently named marine species richness. Log_10_ number of papers as a function of the log_10_ total number of valid species names contained in the World Registry of Marine Species. Solid lines are fitted Generalized Additive Models and dashed lines 95% confidence limits. Ecosystems are plotted individually in panels A–D.

The number of papers for different classes in the four different ecosystems indicated that research has been highly uneven with respect to the taxa investigated, with a very small number of classes having being the subject of the bulk of the research effort to date (Table 1). The number of research papers within each class for all four ecosystems was positively related to the total number of species recorded in the World Registry of Marine Species (WoRMS) [16] (Fig. 3). However, this relationship was relatively weak (R^2^ values ranged from 0.28 to 0.52), with some classes showing considerably greater research effort relative to their known species richness and others much lower (Fig. 3).

**Figure 4:**
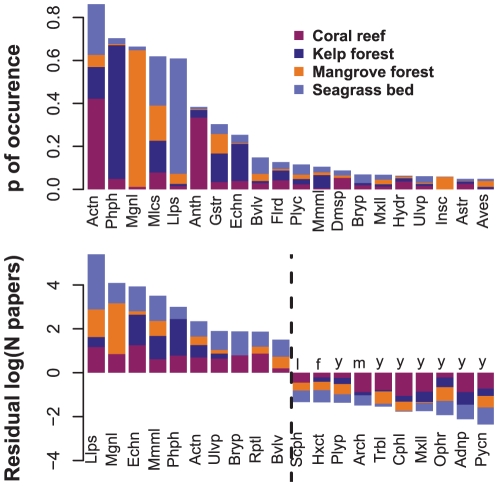
Relative research effort among taxonomic classes. The top 20 ranked classes based on the probability of occurrence in the literature from the four different ecosystems (upper graphs) and the 10 most studied and 10 least-studied classes based on species richness corrected research effort (lower graph)(i.e., deviations from fitted GAMM's shown in Fig. 3.). Annotations over the 10 least studied classes indicate the likelihood of that taxon being present in any of the four ecosystems: y – known to occur, l – likely to occur, m – might occur, and f – relatively few individuals have been reported to occur therein. Taxonomic class abbreviations are as follows: Actn (Actinopterygii), Adnp (Adenophorea), Anth (Anthozoa), Arch (Arachnida), Astr (Asteroidea), Aves (Aves), Bryp (Bryopsidophyceae), Bvlv (Bivalvia), Cphl (Cephalopoda), Dmsp (Demospongiae), Echn (Echinoidea), Flrd (Florideophyceae), Gstr (Gastropoda), Hxct (Hexactinellida), Hydr (Hydrozoa), Insc (Insecta), Llps (Liliopsida), Mgnl (Magnoliopsida), Mlcs (Malacostraca), Mmml (Mammalia), Mxll (Maxillopoda), Ophr (Ophiuroidea), Phph (Phaeophyceae), Plyc (Polychaeta), Plyp (Polyplacophora), Pycn (Pycnogonida), Rptl (Reptilia), Scph (Scaphopoda), Trbl (Turbellaria), Ulvp (Ulvophyceae).

Summed across all ecosystems, the Actinopterygii (fishes) were the most frequently studied class, with some 1559 papers (Table 2). Within all four ecosystems, fish research was also a large component. For coral reefs fishes were the subject of 30.6% of research papers (Table 2). For all three of the other ecosystems, fishes were also one of the most studied groups by actual numbers of papers published (Table 2), showing a high probability of occurrence in the literature, as well as when research effort (numbers of papers) was corrected for species richness (Fig. 4).

Research on all four ecosystems was also largely focused on research on their respective habitat-forming classes (e.g. 24.3%, 41.2%, 49.3% and 34.6% of the research papers for coral reefs (Anthozoa), kelp forests (Phaeophyceae), mangrove forests (Magnoliopsida) and seagrass beds (Liliopsida), respectively; Table 2). This dominance of research on habitat-forming classes is reflected in the high probability of occurrence in the respective literature, and remains after correcting research effort for species richness (Fig. 4).

Along with fishes, and the habitat forming taxa, several other classes were well studied across a range of ecosystems. The Echinoidea, which occurred frequently in the coral reef, kelp-forest and seagrass literature, were also studied more than expected given their species richness (Fig. 4). While the Malacostraca and Gastropoda contributed substantially in terms of total numbers of papers (Table 2) these groups were apparently studied relatively less than expected given their species richness (Fig. 4). In contrast, there are several classes (e.g. Mammalia, Reptilia and Ulvophyceae) that, while not contributing much to the literature in terms of total numbers of papers, have clearly been studied relatively more than expected given their species richness (Fig. 4).

A wide range of classes also appears to have been studied relatively less than expected given their species richness (Fig. 4). Many of these belong to the phylum Arthropoda (Arachnida, Cephalocaridae, Maxillipoda and Pygnogoda), but also included here was a group of nemertean worms (Adenophorea), brittle stars (Ophiuroidea) and glass sponges (Hexactinellida). For many taxonomic classes, few (<10) or no research papers were found for any of these ecosystems (Table 3).

**Table 3:** Classes of marine Phyla (or Division) occurring in the World Registry of Marine Species (WoRMS) with less than 10 occurrences in the *Web of Science*® indexed literature for any of the four ecosystems.

Phylum/Division	Class
Acoelomorpha	Acoela (flatworms, *407*)
Annelida	Clitellata (segmented worms, *444*)
Acanthocephala	Eoacanthocephala & Palaeacanthocephala (types of parasitic worms, *35 & 392*)
Arthropoda	Arachnida (Spiders, mites, *1280*), Branchiopoda (fairy shrimp, *96*), Cephalocarida (Horseshoe shrimps, 12), Remipedia (primitive blind crustaceans, *24*), Chilopoda (centipedes, *56*), Diplopoda (millipedes, *11*), Pauropoda (centipede-like, *8*), Pycnogonida (Sea spiders, 1380), Merostomata (Horseshow crabs, 4), Symphyla (centipede-like, 5)
Bacillariophyta	Coscinodiscophyceae & Fragilariophyceae (diatoms, *615* & *164*)
Brachiopoda	Craniata & Lingulata (inarticulate lamp shells, *19* & *25*), Rhynchonellata (articulate lamp shells, *24*)
Bryozoa	Phylactolaemata & Stenolaemata (moss animals, *79 & 207*)
Cephalorhyncha	Loricifera (girdle wearers or loriciferans, *26*), Nematomorpha (horsehair worms, *5*), Priapulida (cactus worms, *20*), Kinorhyncha (Mud dragons, *162*)
Chaetognatha	Sagittoidea (arrow worms, *208*)
Charophyta	Klebsormidiophyceae (type of green algae, *5*)
Chlorarachniophyta	Chlorarachniophyceae (type of algae, *8*)
Chlorophyta	Charophyceae (charophytes, *400*), Nephroselmidophyceae (*21*), Pedinophyceae (*17*), Pleurastrophyceae (*5*), Prasinophyceae (*125*), Trebouxiophyceae (*20*) (all various types of algae)
Chordata	Larvacea (pelagic tunicates, *83*), Cephalaspidomorphi (lampreys and jawless fishes, *17*), Myxini (hagfish, *75*), Holocephali (*46*), Sarcopterygii (coelacanths, lungfishes, tetrapods, *2*), Leptocardii (lancelets, *33*)
Cnidaria	Polypodiozoa (parasitic, *1*), Staurozoa (stalked jellyfish, *48*), Cubozoa (box jellyfish, *41*)
Cryptophyta	Cryptophyceae (brownish-green protozoa-like algae, *34*)
Craspedophyta	Craspedophyceae (*14*)
Ctenophora	Nuda (comb jellies, lacking tentacles, *23*)
Cycliophora	Eucycliophora (*2*)
Echiura	Echiuroidea (spoon worms, *201*)
Hemichordata	Enteropneusta (acorn worms, *99*), Pterobranchia (worm-like, *25*)
Heterokontophyta	Mediophyceae (algae, *1*)
Mesozoa	Orthonectida (orthonectids, *30*), Rhombozoa (parasitic dicyemids, *95*)
Mollusca	Caudofoveata & Solenogastres (both small, worm like shell-less, *134*), Cephalopoda (Octopus, squid, cuttlefish, nautiluses, *939*), Monoplacophora (monoplacophorans, *30*), Polyplacophora (chitons, *984*), Scaphopoda (tusk shells, *564*)
Myxozoa	Microsporea & Myxosporea (small parasites, *142 & 318)*)
Nematoda	Secernentea (roundworms, 277)
Nemertina	Anopla, Enopla (types of ribbon worms, *1365*)
Ochrophyta	Bicosoecophyceae (27), Bolidophyceae (*2*), Dictyochophyceae (*137*), Eustigmatophyceae (*6*), Phaeothamniophyceae (*3*), Pelagophyceae (12), Pinguiophyceae (*6*), Placidiophyceae (*1*), Raphidophyceae(*19*), Schizocladiophyceae (*1*), Synurophyceae(*32*), Xanthophyceae(*41*) (types of algae)
Platyhelminthes	Turbellaria (free-living flatworms, *1571*), Cestoda (tapeworms, *558*)
Porifera	Calcarea (calcareous sponges, *716*), Hexactinellida (glass sponges, *591*)
Pteridophyta	Filicopsida (ferns, *3*)
Rhodophyta	Bangiophyceae (*245*), Compsopogonophyceae (*53*), Rhodellophyceae (*4*), Stylonematophyceae (*10*), Rhodophyceae (*151*) (types of red algae)
Rotifera	Eurotatoria & Pararotatoria (rotifers, *223* & *3*)
Sipuncula	Phascolosomatidea Sipunculidea (peanut worms, *59 & 110*)
Tardigrada	Heterotardigrada (*402*) & Eutardigrada (*627*) (water bears)

List is limited to classes recorded as occurring in “marine” or “brackish” environments in WoRMS. Numbers in *italics* indicate the number of accepted species, subspecies or variants as recorded in WoRMS.

## Discussion

In the four ecosystems studied, a majority of the research has concentrated on only a few groups of organisms. Although there was a positive relationship between (named) global marine species richness and research effort among different taxonomic classes, some groups were greatly overrepresented in the scientific literature relative to their named species richness while others were greatly underrepresented. To some extent reflective of the economic or perceived ecological significance of some taxa over others, this imbalance suggests that key taxa and ecological processes may be poorly understood. Given that known diversity must also depend to come extent on previous research effort, some of the groups reported here as being understudied are likely to be more diverse than currently recognized. Indeed, undiscovered species of fishes (Pisces) are estimated to be 20–30% of the known fauna, whereas less studied groups such as sponges and platyhelminthes are in the order of 200–300% and nematodes more than an order of magnitude more [26]. If less-studied groups contain more undiscovered species, the extent of the bias we report may be underestimated. Further, if ecological papers (which likely do not list taxonomic names in the text fields considered here - keywords, title and abstract) are biased towards better studied taxa, the disparity between well-studied and poorly-studied taxa may be even more pronounced.

### Variation among taxa in research effort

Among the better-studied groups in all four ecosystems were the dominant habitat-forming organisms: corals, kelps, mangroves and seagrasses. These species provide the physical structure that allows them to host associated species as well as provide other ecosystem goods and services [6], [7], [8], [27], [28], [29], [30], [31]. Given the importance of these taxa to the functioning of these ecosystems, it is expected and appropriate they have been well studied.

Other groups of well-studied taxa were those that are commercially important, large and conspicuous, or which perform other key functional roles in some ecosystems. For all ecosystems, fishes were one of the best-studied taxa. This was true even when the species richness of this group was taken into account. Again, this emphasis on fishes is not surprising, as they are the most widely distributed and diverse vertebrates on earth [32]. Fish are also of great economic value as food and because of their aesthetic value to tourists. Fishes also contribute to critical processes in ecosystem function with some considered keystone species (e.g. [33]). Aside from fishes, other potentially commercially-important taxa that have been frequently studied, including gastropods, bivalves, malacostracan crustaceans and echinoids are important herbivores in a range of ecosystems [34], [35], [36]. Other well-studied taxa (especially relative to their overall diversity) are other large and conspicuous groups, such as mammals and reptiles. These groups also tend to have high conservation value often being endangered or threatened or playing key functional roles [37], and high economic value in tourism and artisanal fisheries.

Greater than average research effort afforded to some taxonomic groups may be appropriate given their economic and ecological importance. Indeed, even for the most studied taxa, the fishes, some 21% of species across all habitat types remain to be described globally and at fine spatial scales (350 km^2^ spatial resolution) only a tiny fraction of the world's oceans have their fish fauna more than 80% described [38]. Therefore, it seems likely that even in well-studied classes (such as fishes) much of our knowledge is sparse and unevenly distributed among their constituent species.

Across all four ecosystems, a large number of classes were not represented in the literature or have received very little research attention relative to their known diversity. The extent to which these groups are truly understudied depends largely on their actual prevalence in these ecosystems. We do not have information on species richness and abundance for all potentially important taxonomic groups for any of these ecosystems and thus our analysis is based necessarily on named global marine taxa (as currently recorded by WoRMS). There is no doubt that some of these groups remain understudied in some ecosystems because they are not a dominant feature there, and/or the bulk of their diversity is found elsewhere. For example, one of the least-studied groups among all four ecosystems was a class of Porifera known as the glass sponges (Hexactinellida) which, while relatively numerous, are most common in deepwater and the Antarctic [39] and are largely lacking in the ecosystems studied here. Some other groups that remain poorly studied in these shallow water ecosystems may also be largely absent. Using information available online, we attempted to provide an indication of whether each class is likely to be represented in any one of the ecosystems considered here (Fig. 4, Table S1). However, reliable information on habitat affiliations of marine taxa is still largely unavailable for many relatively understudied taxa. A detailed examination of the geographic and ecological distribution of each group would help to elucidate the extent to which understudied groups are in fact underrepresented in these ecosystems relative to their potential importance.

While some taxa may be justifiably ignored in these four ecosystems (e.g. if they do not commonly occur there), some highly speciose groups are underrepresented in the literature and may be very important in these ecosystems. Compared to their described diversity, several classes of Arthropoda have been poorly studied in all four ecosystems, and are likely to be prevalent in some (Fig. 4, Table S1). In terrestrial ecosystems, arthropods are highly diverse [40] playing many functional roles [41]. Similar patterns and breadth of ecological function are likely to occur in marine environments. In addition to some of the Arthropoda, several other groups of benthic invertebrates were also understudied with respect to their described diversity. Benthic invertebrates more generally are likely to play an important role in many ecosystems as they span all trophic levels, are important food sources at higher trophic levels and perform crucial roles in bioturbation, oxygenation, nutrient cycling and transport and processing of pollutants [42].

### Variation among ecosystems in taxonomic diversity of research

Considerable differences were evident among these four ecosystems in terms of the total (and expected) species richness represented in their respective literatures and their taxonomic distinctness. The coral reef literature reported on more species than other literatures but also had the lowest level of taxonomic distinctness. Taxonomic distinctness is a measure of the average distance between all pairs of species in the taxonomic tree and low values suggest that the bulk of research is on a limited range of taxonomic groups. Without complete community inventories for these ecosystems, it is impossible to know if the patterns represented by the coral reef literature accurately reflect their community structure, or are a result of particularly biased efforts in research on coral reefs (e.g. a bias favouring corals and fishes, because other groups are much harder to enumerate and identify, or because of a bias in research funding). If the patterns observed reflect greater research bias on coral reefs compared to other ecosystems, this suggests that our capacity to understand and model these complex ecosystems is less than in others. Further, the bias towards research on a limited subset of coral reef taxa is greatest in recent literature, suggesting that the situation is getting worse. This is likely in part because the earliest period that we examined was considerably longer (>40 years) than the other two, and as such involved several generations of scientists, potentially with more varied expertise. However, despite their differences in length, these categories were defined by having similar numbers of publications. The progressive shortening of these periods and the decrease in taxonomic distinctness thus indicate increased research effort is more focused on corals and fishes.

The large taxonomic biases in research effort observed here are likely to be exacerbated, in part, by the dynamics of the current research funding culture. As more research is done on a particular group (e.g. corals and fishes), these groups begin to assume the status of model systems, whereby future research can be leveraged off previous advances in knowledge. While the use of model systems in this way can find favour with reviewers of grant applications and funding agencies, and can have some advantages in terms of building specialist knowledge of particular parts of ecosystems, given finite resources more general knowledge of these systems must be traded off. Such trade-offs may be acceptable where the knowledge gained is applicable to other components of the ecosystem of interest. Such equivalency, however, is not always safe to assume [43], nor easy to test, where data on key species and/or functional groups do not exist. Biases in research effort are also likely to arise when taxonomic expertise is limited and focused on particular taxa. It is well documented that taxonomic effort does not tend to reflect true biological diversity [44] and certain groups are more likely to get identified (and are thereby studied more readily) than others, simply due to their being more taxonomists working on that group.

### Future Allocation of Research Effort Among Taxa

Our results indicate an imbalance in research effort among major taxonomic groups for the four marine ecosystems examined. However, it remains difficult to assess the best way to allocate limited research capacity towards future efforts. Research programs driven solely by the immediate needs of management risk overlooking new insights and opportunities [45]. Conversely, research focused beyond these immediate concerns risk being perceived as irrelevant [45].

Conservation status (or success) is often measured by monitoring target taxa thought to act as indicators of ecosystem health and/or function or biodiversity as a whole. Several criteria are important for selecting indicator taxa [46], but to apply these criteria effectively, considerable ecological knowledge is required, thus limiting the choice of possible indicators to a small range of taxa that may or may not prove adequate for monitoring the health of ecosystems. Likewise, biological surrogates (typically well known and easy to survey groups) are often used as a means of assessing biodiversity patterns without having to resort to exhaustive surveys [47]. However, cross-taxon surrogates are rarely effective, and research focused on only a few select taxa is unlikely to provide good predictors of the wider taxonomic diversity or functioning of an ecosystem [10], [43], [48]. While research should, and will, continue on many well-studied groups, in our opinion, if we are to improve the effectiveness of ecosystem-based management and conservation, more effort needs to be directed towards understanding a broader range of taxa and their interactions.

## Supporting Information

Table S1 **Complete list of taxonomic classes for which there was at least 1 occurrence in the literature indexed in** ***Web of Science*** **® for any of the four ecosystems.**
(DOC)
