# Supplementary material for: Differences among Major Taxa in the Extent of Ecological Knowledge across Four Major Ecosystems
Source: PLoS One. 2011 Nov 2;6(11):e26556. doi: 10.1371/journal.pone.0026556 (PMC3206803; doi:10.1371/journal.pone.0026556)
Supplement: Table S1 — Complete list of taxonomic classes for which there was at least 1 occurrence in the literature indexed in Web of Science ® for any of the four ecosystems. (DOC) [file pone.0026556.s001.doc]

**Table S1. Numbers of research papers by taxonomic class for four marine ecosystems.**

| **Phylum/Division** | **Class** | **Common names** | **CR** | **KF** | **MF** | **SB** | **Total** | **Present** |
| --- | --- | --- | --- | --- | --- | --- | --- | --- |
| Chordata | Actinopterygii | Ray-finned fishes | **1256** | 29 | 31 | **243** | 1559 | y |
| Cnidaria | Anthozoa | Sea anemones, corals (various) | **994** | 7 | 2 | 11 | 1014 | y |
| Angiospermophyta | Liliopsida | Seagrasses | 45 | 2 | 26 | **553** | 626 | y |
| Arthropoda | Malacostraca | Crabs, lobsters, shrimp, krill, amphipods, isopods | 233 | 29 | **90** | 236 | 588 | y |
| Angiospermophyta | Magnoliopsida | Mangroves | 34 | 0 | **350** | 17 | 401 | y |
| Ochrophyta | Phaeophyceae | Brown algae (including kelp) | 145 | **122** | 3 | 28 | 298 | y |
| Mollusca | Gastropoda | Snails, slugs | 102 | 26 | 50 | 47 | 225 | y |
| Echinodermata | Echinoidea | Sea urchins, sand dollars | 115 | **34** | 2 | 39 | 190 | y |
| Porifera | Demospongiae | Sponges | 159 | 0 | 6 | 24 | 189 | y |
| Mollusca | Bivalvia | Bivalves | 86 | 2 | 18 | 79 | 185 | y |
| Rhodophyta | Florideophyceae | Red algae | 124 | 9 | 4 | 33 | 170 | y |
| Annelida | Polychaeta | Segmented worms | 68 | 5 | 11 | 49 | 133 | y |
| Cnidaria | Hydrozoa | Hydrozoans | 109 | 3 | 4 | 4 | 120 | y |
| Chlorophyta | Bryopsidophyceae | Green algae (various) | 57 | 2 | 0 | 41 | 100 | y |
| Echinodermata | Asteroidea | Starfish | 78 | 2 | 1 | 11 | 92 | y |
| Chlorophyta | Ulvophyceae | Green algae (sea lettuce) | 46 | 2 | 3 | 31 | 82 | y |
| Bryozoa | Gymnolaemata | Moss animals | 77 | 1 | 0 | 2 | 80 | y |
| Arthropoda | Maxillopoda | Barnacles, copepods | 40 | 2 | 12 | 23 | 77 | y |
| Chordata | Mammalia | Mammals | 15 | 12 | 7 | 27 | 61 | y |
| Chordata | Ascidiacea | Sea squirts | 30 | 2 | 0 | 5 | 37 | y |
| Arthropoda | Insecta | Insects | 0 | 0 | 32 | 1 | 33 | y |
| Echinodermata | Holothuroidea | Sea cucumbers | 20 | 0 | 0 | 13 | 33 | y |
| Chordata | Aves | Birds | 4 | 2 | 15 | 10 | 31 | y |
| Chordata | Reptilia | Reptiles (sea snakes, turtles, crocodiles) | 22 | 0 | 2 | 7 | 31 | y |
| Ctenophora | Tentaculata | Comb jellies (with tentacles) | 29 | 0 | 0 | 0 | 29 | y |
| Chordata | Elasmobranchii | Sharks, rays, skates | 22 | 0 | 1 | 5 | 28 | y |
| Platyhelminthes | Trematoda | Flukes | 27 | 0 | 1 | 0 | 28 | y |
| Platyhelminthes | Monogenea | Ectoparasitic flatworms | 20 | 0 | 1 | 1 | 22 | y |
| Nematoda | Adenophorea | Roundworms | 8 | 0 | 7 | 3 | 18 | y |
| Cnidaria | Scyphozoa | True jellyfish | 16 | 0 | 0 | 0 | 16 | y |
| Echinodermata | Ophiuroidea | Brittle stars and basket stars | 15 | 0 | 0 | 1 | 16 | y |
| Chordata | Thaliacea | Salps and relatived (all free-floating) | 1 | 0 | 0 | 12 | 13 | y |
| Bacillariophyta | Bacillariophyceae | Pennate diatoms | 4 | 0 | 3 | 4 | 11 | y |
| Arthropoda | Ostracoda | Seed shrimp | 5 | 0 | 2 | 4 | 11 | y |
| Chlorophyta | Chlorophyceae | Green algae (various) | 6 | 1 | 1 | 2 | 10 | y |
| Echinodermata | Crinoidea | Sea lilies, feather stars | 6 | 0 | 4 | 0 | 10 | y |
| Nematoda | Secernentea | Roundworms | 8 | 0 | 1 | 0 | 9 | l |
| Annelida | Clitellata | Segmented worms | 4 | 0 | 3 | 1 | 8 | l |
| Bryozoa | Stenolaemata | Moss animals | 4 | 0 | 0 | 3 | 7 | y |
| Mollusca | Polyplacophora | Chitons | 6 | 0 | 0 | 1 | 7 | y |
| Platyhelminthes | Turbellaria | Free-living flatworms | 2 | 1 | 0 | 4 | 7 | y |
| Porifera | Calcarea | Calcareous sponges | 7 | 0 | 0 | 0 | 7 | y |
| Rhodophyta | Rhodophyceae | Red algae | 5 | 0 | 0 | 1 | 6 | y |
| Bacillariophyta | Coscinodiscophyceae | Diatoms | 2 | 0 | 3 | 1 | 6 | y |
| Arthropoda | Arachnida | Spiders, mites | 1 | 0 | 4 | 1 | 6 | m |
| Chaetognatha | Sagittoidea | Arrow worms | 3 | 0 | 0 | 2 | 5 | u |
| Pteridophyta | Filicopsida | Ferns | 0 | 0 | 4 | 0 | 4 | u |
| Cryptophyta | Cryptophyceae | Brownish-green protozoa-like algae | 3 | 0 | 0 | 1 | 4 | m |
| Ctenophora | Nuda | Comb jellies (lacking tentacles) | 4 | 0 | 0 | 0 | 4 | l |
| Mollusca | Cephalopoda | Octopus, squid, cuttlefish, nautiluses | 0 | 0 | 0 | 4 | 4 | y |
| Platyhelminthes | Cestoda | Tapeworms | 3 | 0 | 1 | 0 | 4 | m |
| Porifera | Hexactinellida | Glass sponges | 4 | 0 | 0 | 0 | 4 | f |
| Chordata | Sarcopterygii | Coelacanths, lungfishes, tetrapods | 3 | 0 | 0 | 0 | 3 | f |
| Rotifera | Eurotatoria | Rotifers | 2 | 0 | 1 | 0 | 3 | y |
| Bacillariophyta | Fragilariophyceae | Diatoms | 0 | 0 | 2 | 0 | 2 | y |
| Ochrophyta | Pelagophyceae | Algae | 0 | 0 | 0 | 2 | 2 | u |
| Ochrophyta | Raphidophyceae | Algae | 1 | 0 | 0 | 1 | 2 | l |
| Acoelomorpha | Acoela | Flatworms | 0 | 0 | 0 | 2 | 2 | l |
| Arthropoda | Pycnogonida | Sea spiders | 2 | 0 | 0 | 0 | 2 | y |
| Cephalorhyncha | Priapulida | Cactus worms | 2 | 0 | 0 | 0 | 2 | l |
| Chordata | Leptocardii | Lancelets | 0 | 0 | 0 | 2 | 2 | l |
| Cnidaria | Cubozoa | Box jellyfish | 2 | 0 | 0 | 0 | 2 | y |
| Mollusca | Scaphopoda | Tusk shells | 1 | 1 | 0 | 0 | 2 | l |
| Myxozoa | Myxosporea | Microscopic parasites | 2 | 0 | 0 | 0 | 2 | l |
| Sipuncula | Sipunculidea | Peanut worms | 1 | 0 | 0 | 1 | 2 | l |
| Heterokontophyta | Mediophyceae | Algae | 1 | 0 | 0 | 0 | 1 | l |
| Chlorophyta | Prasinophyceae | Green algae (various) | 1 | 0 | 0 | 0 | 1 | l |
| Chlorophyta | Trebouxiophyceae | Green algae (various) | 0 | 0 | 0 | 1 | 1 | l |
| Ochrophyta | Bicosoecophyceae | Algae | 1 | 0 | 0 | 0 | 1 | u |
| Ochrophyta | Synurophyceae | Synurids(algae) | 0 | 0 | 1 | 0 | 1 | u |
| Ochrophyta | Xanthophyceae | Yellow-green algae | 0 | 0 | 1 | 0 | 1 | l |
| Arthropoda | Merostomata | Horseshoe crabs | 1 | 0 | 0 | 0 | 1 | l |
| Arthropoda | Cephalocarida | Horseshoe shrimps | 0 | 0 | 0 | 1 | 1 | l |
| Brachiopoda | Craniata | Lamp shells (inarticulate) | 1 | 0 | 0 | 0 | 1 | m |
| Brachiopoda | Rhynchonellata | Lamp shells (articulate) | 1 | 0 | 0 | 0 | 1 | m |
| Cephalorhyncha | Kinorhyncha | Mud dragons | 0 | 0 | 0 | 1 | 1 | l |
| Echiura | Echiuroidea | Spoon worms | 1 | 0 | 0 | 0 | 1 | u |
| Mesozoa | Rhombozoa | Dicyemids (parasitic) | 1 | 0 | 0 | 0 | 1 | m |

CR – Coral reefs, KF – Kelp forests, MF – Mangrove forests, SB – Seagrass beds. Phylum and Class information was obtained from the World Registry of Marine Species (WoRMS [16]). Only classes with at least 1 occurrence in the literature indexed in *Web of Science*® for any of the four ecosystems are shown. The two most studied classes for each ecosystem are shown in **bold.** The final column “Pres.” indicates the likelihood of a taxon being present in any of the four ecosystems: y – known to occur there, l – likely to occur, m – might occur, u – unable to find information, and f – relatively few individuals have been reported to occur therein. Common names are not comprehensive but provide examples for the groups; in some cases no common names specific for the group exist and more general common names are provided.
